# The asymmetry of plasma membranes and their cholesterol content influence the uptake of cisplatin

**DOI:** 10.1038/s41598-019-41903-w

**Published:** 2019-04-04

**Authors:** Timothée Rivel, Christophe Ramseyer, Semen Yesylevskyy

**Affiliations:** 1 Laboratoire Chrono Environnement UMR CNRS 6249, Université de Bourgogne Franche-Comté, 16 route de Gray, 25030 Besançon, Cedex France; 2 Department of Physics of Biological Systems, Institute of Physics of the National Academy of Sciences of Ukraine, Prospect Nauky 46, 03028 Kyiv, Ukraine

## Abstract

The composition of the plasma membrane of malignant cells is thought to influence the cellular uptake of cisplatin and to take part in developing resistance to this widespread anti-cancer drug. In this work we study the permeation of cisplatin through the model membranes of normal and cancer cells using molecular dynamics simulations. A special attention is paid to lipid asymmetry and cholesterol content of the membranes. The loss of lipid asymmetry, which is common for cancer cells, leads to a decrease in their permeability to cisplatin by one order of magnitude in comparison to the membranes of normal cells. The change in the cholesterol molar ratio from 0% to 33% also decreases the permeability of the membrane by approximately one order of magnitude. The permeability of pure DOPC membrane is 5–6 orders of magnitude higher than one of the membranes with realistic lipid composition, which makes it as an inadequate model for the studies of drug permeability.

## Introduction

Although the structure and major properties of the cell membranes are considered to be known since the introduction of the fluid-mosaic model^1^, there are still many open questions concerning membrane composition, permeability and mechanical properties. The continuous increase of computer power has stimulated the rapid development of computational approaches to the modeling of realistic cell membranes using both atomistic and coarse-grained molecular dynamics (MD) techniques^2–8^. In particular, recent works address such phenomena as lateral heterogeneity of the membranes (lipid rafts and micro-domains, lipid sorting)^2,3^; asymmetry of the lipid composition and cholesterol content between the monolayers^9^; membrane curvature and its influence on the physical properties of bilayer^10^.

The existence of lipid rafts or micro/nano-domains in the membranes is still a debated concept. It is shown that cholesterol (CHL) and sphingomyelin lipids (SM) form regions of rigid liquid-ordered phase (Lo) *in vitro* in giant unilamellar vesicles (GUVs), in liposomes or in deposited lipid structures^11–13^. The rafts were also extensively studied *in silico*^11,14–16^. However, there are difficulties in observing the rafts in real cells, which are usually attributed to their small life time or to their size, which appears to be much smaller in real cells in comparison to artificial membranes^14,17,18^. Despite the fact that the rafts are not observed directly in living cells, the influence of cholesterol and cholesterol-rich microdomains on the membrane rigidity, on permeability for small molecules and on the functioning of the membrane proteins are of great interest^19–22^.

An asymmetry in the lipid composition between the monolayers of the plasma membrane is now recognized as an important factor of membrane functioning. The composition of the membrane leaflets is highly uneven^13,23,24^ and is actively maintained by a group of proteins – the flippases and floppases. This results in an extracellular leaflet composed mostly of phosphatidylcholine (PC) and SM and a cytoplasmic leaflet enriched in phosphatidylserine (PS) and phosphatidylethanolamine (PE). The transversal distribution of CHL is subject to debates. It has been observed experimentally^25–27^ that CHL resides mostly in the cytoplasmic leaflet. On the other hand, the affinity of cholesterol to SM has been shown experimentally^18^ and *in silico* simulations also tend to show an increased distribution of CHL to the outer leaflet^9,28,29^. Although more and more computational studies are trying to mimic an actual asymmetric lipid content of the membrane monolayers^4,5,7,10^, we are not aware of any works which are addressing the question of the influence of this asymmetry on the permeability of the membranes to drugs and small molecules.

Besides the asymmetry of the membrane, its curvature also plays an important role in many cellular processes^26,30^. The curvature was recently shown to induce changes in the distribution of cholesterol in the membrane, in the thickness of its leaflets and in the order parameter of the lipid tails^10^ but the studies of the influence of curvature on the passive diffusion of drugs and small molecules through the membrane are scarce.

Accounting for membrane asymmetry and realistic lipid composition in MD simulations is especially important due to the fact that this composition changes significantly in malignant cells. Since 1989 it is known that some cancer cells expose PS on the extracellular leaflet of their plasma membranes, while this anionic lipid is predominantly located in the intracellular leaflet in the normal cells^31,32^. Subsequent studies using flow cytometry after labeling by annexin V confirmed these findings^33–35^. It was also shown that exposure of PS in cancer cells is not an artifact caused by the presence of apoptotic cells in the sample^36^. PS exposure was also detected in the vasculature of the tumors^37,38^. Table S1 in Supplementary Information summarizes existing literature about PS exposure in different cancers and cancer cell lines. It is evident that the outer monolayer of cancer cells contains on average ~7 times more PS than the normal control cells. These findings resulted in a new approach in targeting tumors by selective recognition of exposed PS^35,39–42^ or related redistribution of PE^43^. Despite the growing importance of this field we have found little research where *in silico* modeling of the membranes of cancer cells was performed^6^.

MD simulations provide a unique opportunity for studying the diffusion of drugs and small molecules through realistic model membranes in atomic details, although the methodology of computing permeabilities from MD simulations is still subject to debate. The most widespread method of computing permeabilities is the inhomogeneous solubility-diffusion (ISD) model^44,45^, which accounts for transversal heterogeneity of the membrane by means of non-uniform diffusion coefficient of the ligand. The ISD model was used successfully in many studies^46,47^ (see^48^ for extensive review). However, a number of publications report consistent difficulties in computing free energy profiles and diffusion coefficients and discuss possible improvements in ISD methodology^46,49–53^. One of the major concerns comes from the difficulty of determining equilibrium state of the ligand in the membrane related to extremely slow transitions in bilayer structure, which occur on the time scale well beyond the capabilities of routine all-atom MD simulations^54^. Another concern is related to computations of diffusion coefficients of the ligands. Anomalous diffusion regimes were recently reported for small molecules in lipid bilayers^55^, which questions the validity of ISD approximation on typical MD time scales. The influence of the long-lived correlations of the restrained solute on diffusion coefficients in the membrane was also suggested as a possible source of errors in ISD^56^.

There is a consensus that obtaining precise quantitative values of permeabilities from MD simulations is an extremely complex task. Particularly, comparing permeabilities of different ligands in MD simulations is notoriously difficult and currently hampers wide usage of membrane simulations in evaluation of drug candidates. However, MD simulations could still be used to compare the permeabilities of the same ligand in different environments on the semi-quantitative level. In this case the very similar systematic errors apply to all MD simulations, which make them comparable to each other and to available experimental trends despite the fact that exact values of permeabilities are unlikely to be sufficiently accurate.

Permeability of the membranes of normal and cancer cells of the anti-tumor drugs, which are currently available on the market or in the clinical studies pipeline, is of great interest. It is obvious, however, that some well-known and widely used drug should be studied first to show that the difference in permeability between normal and cancer cell membranes exists. Cisplatin (c*is*-diamminedichloroplatinum(II)) is a good candidate for such a study. It is one of the oldest and the most widely used anti-tumor agents, which is adopted for treatment of a wide range of cancers^57^. While its action, which leads to cell death by apoptosis or necrosis by means of DNA damage and redox stress, is well studied (see^58,59^ for reviews), the mechanism of its transport through the membrane is not yet understood. There were numerous studies^60–64^ which supported the hypothesis that the copper transporter CTr1 facilitates the transport of cisplatin into the cell. However, more recent research mostly excludes this hypothesis^57,65–68^. Although some studies show a positive correlation between the presence of CTr1 in the membrane and the high transport rate of cisplatin, they do not necessarily show an increase of the cell death rate. This may suggest that cisplatin appears in the cytoplasm in inactivated chemical form after being transported by CTr1^65,69–71^. Currently passive diffusion through the lipid bilayer is considered as the only known way of transport of cisplatin in its active form^62,69,71,72^.

The chemistry of solvated cisplatin is complex^73^. It undergoes numerous reactions such as oligomerization, hydrolysis, hydroxylation, protonation/deprotonation, substitution of the chloride with carbonate ions, sulfur groups or other nucleophile moieties, *etc*.^74–86^. It is shown that the native form of cisplatin has the highest propensity to passively diffuse through the plasma membrane and is the most abundant form in the extracellular medium^87–89^.

It is possible to conclude that atomistic models of cell membranes with realistic lipid composition and cholesterol distribution are of high demand for both normal and cancer cells. The computations of permeability of such membranes for drugs are of great importance for on-going attempts of targeting the cancer cells by their membrane composition. Moreover, the methodology of computing the permeability coefficients is well-established and tested. Despite this interest there are no dedicated works which are focused on computing permeabilities in normal and cancer cell membranes for common anti-tumor drugs. There are also very few systematic attempts to compare permeabilities of the membranes with different cholesterol content in complex model membranes.

In this work we computed permeabilities of realistic model membranes of normal and cancer cells for the widely used anti-cancer drug cisplatin. We also compared permeabilities in the membranes with different cholesterol content ranging from 0% to 33% molar ratio. It is shown that the cancer cell membrane is approximately one order of magnitude less permeable to cisplatin in comparison to the membrane of the normal cell. We discuss possible reasons for this difference in details and compare obtained results with available experimental data. We use the ISD methodology to compare permeabilities of cisplatin in the membranes of different compositions with the same simulation setup and environmental conditions. We are aware about shortcomings of this technique and thus will interpret obtained permeability data obtained on the semi-quantitative level only.

## Methods

### Model membranes

We used the model of realistic plasma membrane, referred to as “normal membrane” hereafter, developed in our previous work^10^. In this model the asymmetry of real mammalian plasma membranes is taken into account – the outer monolayer is enriched in sphingomyelin (SM) and 1,2-dioleoyl-sn-glycero-3-phosphocholine (DOPC), while the inner monolayer is enriched in 1,2-dioleoyl-sn-glycero-3-phosphoethanolamine (DOPE) and 1,2-dioleoyl-sn-glycero-3-phospho-L-serine (DOPS).

**Figure 1:**
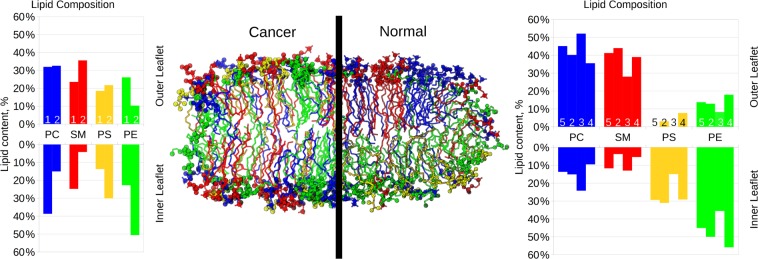
Snapshots of the simulated systems for cancer and normal membrane models. DOPC is shown in blue, SM in red, DOPS in yellow and DOPE in green. For the sake of clarity cholesterol is not shown. Head groups of lipids are shown as spheres. The histograms show the relative abundance of different lipid species for inner and outer monolayers for each membrane model (normalized for each monolayer). Numbers 1 and 5 correspond to the cancer and normal models presented in this work respectively, model 2 refers to the work by Klähn and Zacharias^6^, model 3 to the work by Ingólfsson *et al*.^4^, model 4 to the 1) data reported by Marquardt *et al*.^26^.

The model of the plasma membrane of a cancer cell, referred as “cancer membrane” hereafter, was built using the same protocol as described in 10 but with increased proportion of DOPS and DOPE in the extracellular leaflet. Figure 1 shows a schematic representation of the normal and cancer membranes along with their lipid compositions and comparison to available experimental data. Table 1 shows the lipid content of the monolayers of normal and cancer membranes. The normal membrane was designed according to well-established lipid content of mammalian erythrocyte membrane^90^. In the case of the cancer membrane the distribution of the lipid species was symmetrized to emphasize the overexpression in PS/PE in the extracellular leaflet. The Slipids force field^91^ was used for lipids, which is one of the best force fields for the membrane systems available today^92^.

**Table 1:** Lipid content (absolute number of molecules) of the monolayers of simulated membranes.

Component	Normal		Cancer	
	Outer	Inner	Outer	Inner
SM (sphingomyelin)	42	12	27	27
PC (1,2-dioleoyl-sn-glycero-3-phosphocholine)	46	14	30	30
PE (1,2-dioleoyl-sn-glycero-3-phosphoethanolamine)	14	46	30	30
PS (1,2-dioleoyl-sn-glycero-3-phospho-L-serine)	0	30	15	15
Cholesterol	51	51	51	51

**Figure 2:**
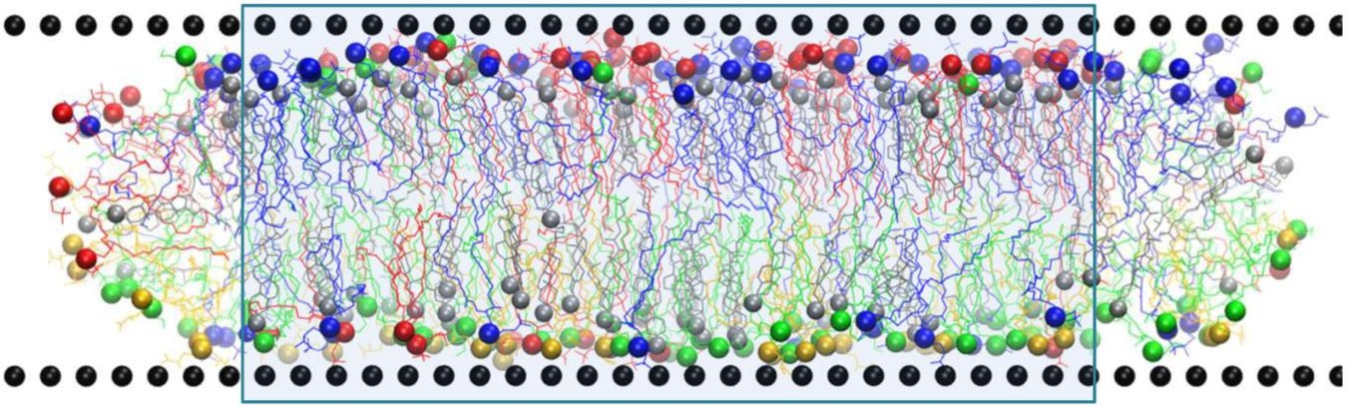
Snapshot of the simulated “normal membrane” system. The wall particles are shown as black spheres. PC lipids are blue, SM are red, PE are green and PS are yellow. Cholesterol molecules are gray. Head groups of lipids and cholesterol are shown as spheres. The central region of the bicelle which is used for analysis is shaded.

The membrane was prepared as a bicelle which is limited by cylindrical caps in XZ plane and forms an infinite bilayer in Y direction (Fig. 2, see also Fig. S1 for a perspective view). This allows the monolayers to relax to their optimal areas by exchanging the lipids with the bicelle caps, which serve as “compensatory reservoirs”. The mixing of the lipids from different monolayers was prevented by artificial repulsive potentials as described in our previous works^9,93,94^. The cholesterol molecules could diffuse freely through the bicelle caps which allows their redistribution between the monolayers during equilibration of the system. This is important because preferred cholesterol content in each monolayer is not known in advance and such mechanism of cholesterol exchange between the monolayers facilitates optimal cholesterol content in equilibrated system. Only the middle of the bicelle, where the bilayer structure is not perturbed by the caps (shaded region in Fig. 2), is used for analysis. This setup was recently used with great success for both atomistic and coarse-grained studies of curved membranes. The technical details are provided in SI and the scheme of interactions in the regions of bicelle caps is shown in Fig. S2. The reader is referred to the works^10,93^ for detailed rationale and discussion of this technique.

Initial bicelle has a cholesterol molar content of 33% relative to the lipids. We also constructed the systems with cholesterol content of 15% and 0% by randomly removing half of the cholesterol molecules in the former and all of them in the later. An equal amount of cholesterol was removed from both monolayers in the case of 15% content.

The reference single-component DOPC membrane was also simulated. This system was also arranged as a bicelle using the same setup except for the fact that no repulsive potential is applied in the caps since the mixing of the monolayers is allowed in this case.

All membranes were equilibrated for at least 200 ns before computing the permeabilities.

### Computations of permeability

The QDsol topology of cisplatin from our previous work was used^95^. This topology reproduces internal flexibility of the coordination bonds of platinum atom accurately by fitting them to the values obtained in *ab initio* quantum dynamics of solvated cisplatin. The ESP point charges and the Van-der-Waals parameters from our previous papers were used^96,97^ where geometry optimization of cisplatin at MP2(FC)/6-31 G(d,p)/LanL2DZ level of theory was performed. This level of theory has been proved to be the most precise and the least time consuming for modelling cisplatin^97^. The force field parameters for cisplatin are available for free as the electronic Supplementary Material in the work^95^.

The potentials of mean force (PMF) of translocation of cisplatin were computed using the umbrella sampling method. In order to improve the sampling of the lateral heterogeneity of the membrane several cisplatin molecules (3 in the case of plasma membrane model and 5 in the case of DOPC model) were placed in equal intervals along the X axis at the bilayer part of the bicelle. We analyzed the nearest neighbors of each ligand during the simulations and concluded that different ligands sample the regions with sufficiently different lipid environments. The details of this analysis are reported in SI and the results are shown in Fig. S3. In our bicelle setup, the lipids preferring large positive curvature could redistribute from the bilayer part of the bicelle to its caps which may change the lipid content of the areas under analysis. It is clear from Fig. S3 that the microenvironments of the ligands are somewhat different from the initial distributions of the corresponding monolayers shown in Table 1. However, these changes are small enough, not systematic and do not lead to the loss of asymmetry in normal membranes or its appearance in cancer membrane. Thus, we conclude that the possible redistribution of the lipids between bilayer part of the bicelle and its caps is not an issue in our setup.

The harmonic biasing potential with the force constant of 1000 kJ/mol/nm^2^ was applied to the centre of mass of each ligand in Z dimension, which is perpendicular to the membrane plane. The potentials were centered at discrete points distributed along Z axis at 0.1 nm intervals. Additional weak flat-bottom potential was applied along the X axis for each ligand to prevent their accidental interaction due to uncontrollable lateral diffusion. This produced 50 to 57 umbrella sampling windows spanning through the whole bilayer along the Z axis. The PMFs were computed as average of all ligands using the weighted histogram technique as implemented in Gromacs. Each window was simulated for at least 16 ns with the last 5 ns being used for sampling.

Table 2 shows the summary of performed simulations with their corresponding simulation times per window and total simulation times.

**Table 2:** Summary of simulations performed.

System Simulation time	DOPC	0% CHL - normal	15% CHL - normal	33% CHL - normal	33% CHL - cancer
Time per umbrella sampling window (ns)	16	16	16	20	20
Total simulation time (µs)	0.8	0.8	0.8	1.0	1.0

A plot showing the convergence of the free energy profile is reported in the Supplementary Information (Fig. S4). The errors of the PMFs were estimated using the bootstrapping method^98^. For each system 400 bootstraps were computed by considering complete histograms as independent data points using the Gromacs WHAM tool.

The diffusion coefficients D of the ligands were computed for each umbrella sampling window using the force correlation method^99^:

$${\rm{D}}({\rm{z}})={({\rm{RT}})}^{2}/{\int }_{0}^{\infty }\,\langle {{\rm{\Delta }}{\rm{F}}}_{{\rm{z}}}({\rm{z}},{\rm{t}}){{\rm{\Delta }}{\rm{F}}}_{{\rm{z}}}({\rm{z}},0)\rangle {\rm{dt}},$$

where *R* is the gas constant, *T* is an absolute temperature, ΔF_z_(z, t) is the difference between the instantaneous force and mean force acting on the permeant at given time *t*. The autocorrelation functions were fitted by single exponential decay prior to integration as implemented in Gromacs tools. Diffusion coefficients of all the ligands were averaged for each window. The trajectory was divided into four equal parts and *D* was computed independently for all of them. The standard deviation of these values is reported as the error of diffusion coefficient and their mean value is used to compute permeabilities.

The permeability coefficients *P* of cisplatin were computed using standard inhomogeneous solubility-diffusion model^99^:

$$P=1/\,{\int }_{{{\rm{z}}}_{1}}^{{z}_{2}}\,R(z)dz,$$

where *R*(*z*) is the local permeation resistance of the membrane at depth *z* expressed as

$$R(z)={e}^{\frac{W(z)}{kT}}/D(z),$$

where *W*(*z*) is the PMF of a given ligand. The integration limits *z*_1_ and *z*_2_ were chosen as ±2.3 nm from the membrane center (the points in the water phase outside the membrane). The value of *P* is insensitive to the choice of these limits since the resistance *R* in water phase is negligible. The errors of *R* and *P* could be evaluated using the error propagation formula based on the standard deviations of *W*(*z*) and of *D*(*z*) as detailed in SI.

### Technical details

All MD simulations were performed in Gromacs^100^ versions 5.1.2 and 2016.1 in NPT ensemble at the pressure of 1 atm maintained by Berendsen barostat^101^ with anisotropic pressure coupling along Y direction only (see^10,93^ for rationale of this choice). The Verlet cutoff scheme is used^102^. Long range electrostatics was computed with the PME method^103^. Velocity rescale thermostat^104^ was used. The temperature of 320 K was used. An integration steps of 1 fs was used in all simulations as required by the “soft” bonds of cisplatin topology^10^. No bonds were converted to rigid constraints (see SI for additional discussion of the validity of used time step settings).

## Results

### Properties of the membranes

**Figure 3:**
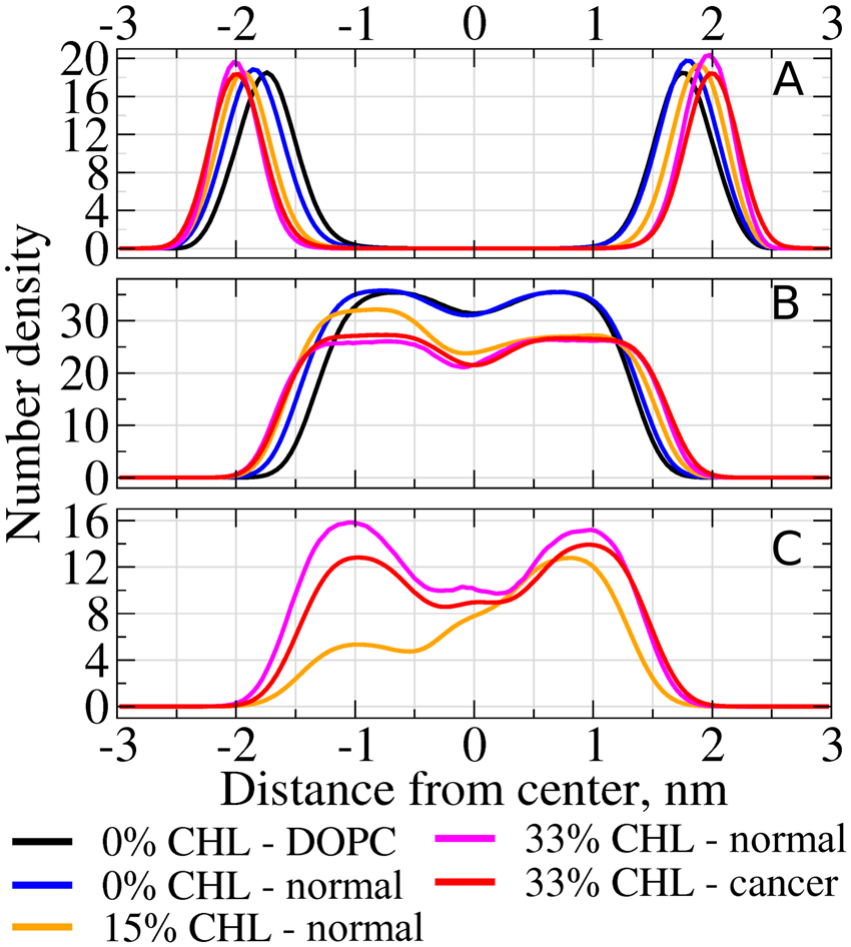
Number densities of the lipid head groups (**A**), lipid tails (**B**) and cholesterol (**C**) for different model membranes. Outer leaflet corresponds to positive distances from the membrane center.

**Figure 4:**
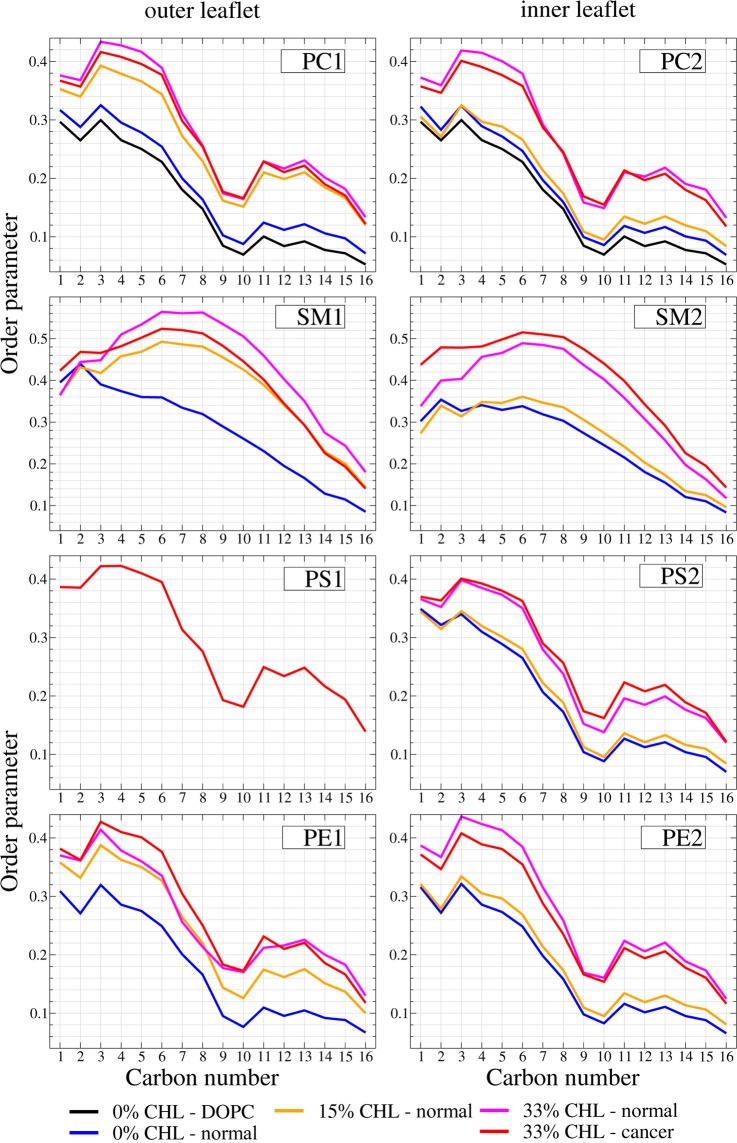
Order parameter of the lipid tails for all studied phospholipid species in the inner and outer monolayers for the different membrane models.

It is well known that cholesterol content changes the physical properties of the membrane significantly, in a way referred to in the literature as the “condensing effect|”^19^. In order to assess these changes in our systems we computed the density profiles of head groups and tails (Fig. 3) and the order parameter of the lipid tails (Fig. 4). These observables were averaged over the last 10 ns of each umbrella sampling windows used for the computation of the PMFs of cisplatin, which sums up to 0.5 µs of total simulation time.

**Figure 5:**
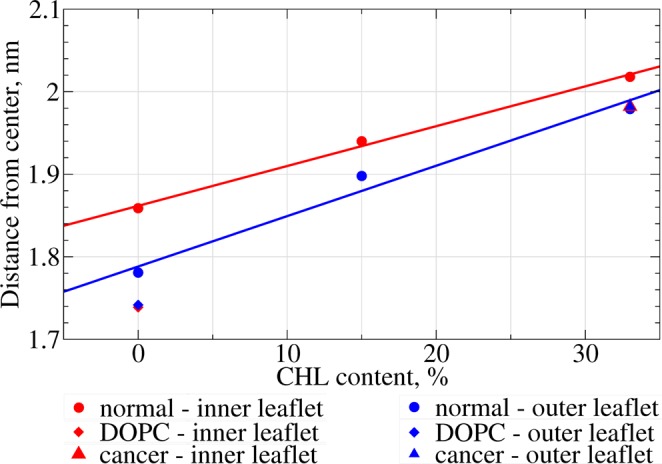
Absolute distance from the center of the membrane to the peak of head groups density for different membrane models as a function of cholesterol content. The lines show linear regression of the corresponding values for inner and outer leaflets.

It is clearly visible from Fig. 3 that the distance between the head groups (the thickness of the membrane) increases with an increase of cholesterol content. This trend is shown as a function of cholesterol content in Fig. 5. The trends for the inner and the outer leaflets of the normal membrane exhibit slightly different slopes but both of them demonstrate perfect linear dependence (the correlation coefficients of linear regression are 0.998 and 0.988 respectively). The pure DOPC bilayer is the thinnest, which is expected taking into account a more compact conformation of its unsaturated lipid tails. The cancer membrane with 33% cholesterol has identical thicknesses of both monolayers which is comparable with the thickness of the outer leaflet of the normal membrane with the same cholesterol content.

The density profiles of cholesterol for normal and cancer membranes at 33% CHL content are comparable. For both normal and cancer membranes, the inner and outer monolayers contain approximately the same number of CHL molecules (the density profiles are symmetric). In our setup the CHL molecules could diffuse freely over the bicelle caps and redistribute between the monolayers. This may explain the visible asymmetry of the cholesterol distribution in the system with 15% of cholesterol (see Discussion for details).

Such redistribution of cholesterol influences the density of the lipid tails. It is evident from Fig. 3 that the density of the tails decreases substantially upon an increase in the cholesterol content. This is expected since CHL molecules intercalate between the tails and increase the total volume of the monolayer. The redistribution of CHL to the outer monolayer correlates with smaller tails density in this monolayer at a 15% cholesterol content.

The order parameters of the lipid tails also show remarkable dependence on cholesterol content. All phospholipids (PC, PE and PS) show very similar trends. The order parameter increases with the increase in cholesterol content in the corresponding monolayer. This general trend is logical and easily explained by the fact that rigid CHL molecules intercalate between the lipid tails and increase their ordering. Such behavior has already been observed in pure 2,3dipalmitoyl-D-glycero-1-phosphatidylcholine (DPPC) and 3-palmitoyl-2-oleyl-D-glycero-1-phosphatidylcholine (POPC) model membranes^15,105^. Asymmetric distribution of cholesterol at 15% is also clearly visible by the different position of the corresponding curve for inner and outer monolayers (the orange curve is much higher for PC1 and PE1 than for PC2 and PE2).

The ordering of PC tails in pure DOPC membrane is lower than the ordering of PC tails in the normal membrane with 0% cholesterol. This is explained by the influence of a more ordered saturated SM tails in the normal membrane.

The differences in order parameter between normal and cancer membrane with 33% of cholesterol are rather small. The tails of PC and PE lipids are less ordered in the cancer membrane while the tails of PS are more ordered in the inner monolayer. This is easily explained by the redistribution of SM lipids. In the cancer membrane SM is distributed evenly between the monolayers, while in the normal membrane most of SM is in the outer leaflet. This leads to a decrease in the ordering in the outer monolayer and its increase in the lower monolayer.

The ordering of the tails of SM lipids themselves follows the same pattern except there is a remarkable difference of the curves with low content of cholesterol (SM1 at 0% and SM2 at 0% and 15%). In the absence of cholesterol the order of saturated SM tails decreases monotonously towards the center of the membrane. In contrast, in the presence of cholesterol the ordering increases and reaches the maximum in the middle of the tail near carbons 7 and 8. This clearly demonstrates the formation of strong SM/CHL complexes. The similar changes of the order parameter were also reported in the case of pure DPPC membranes with an increasing cholesterol content^105^ which is in line with the well-known affinity of cholesterol to saturated lipid tails^11^.

It is thus possible to infer that cholesterol concentration changes the physical properties of the membrane more than the redistribution of the lipids between the leaflets.

### Permeation of cisplatin

**Figure 6:**
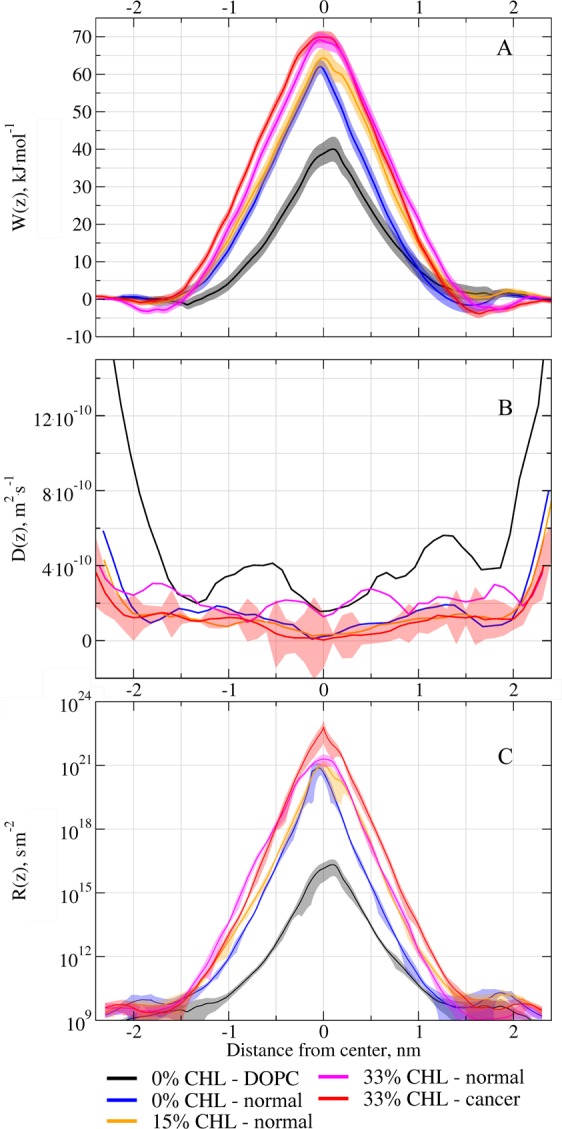
(**A**) The PMFs of cisplatin, *W(z)*; (**B**) the local diffusion coefficient of cisplatin, *D(z)*; (**C**) the local resistance of the membrane *R(z)* (in log scale) for different membrane models. The errors are shown as ribbons for each curve. The errors for diffusion coefficients overlap a lot thus the errors of only one curve are shown in panel B to keep the figure readable. The errors of all other diffusion coefficients curves are shown separately in the Fig. S5.

Figure 6A shows the PMFs of cisplatin permeation through the membranes under study. There is a high energy barrier in the center of the membrane, which is expected for such hydrophilic compound as cisplatin. The shape of the central energy barrier is roughly symmetric because the core region of the membrane is similar in both monolayers except some excess of saturated lipid tails in outer monolayer of asymmetric membrane.

The height of the barrier is the smallest for pure DOPC membrane (~40 kJ/mol) which is comparable to previous results obtained in a pure 1,2-dimyristoyl-sn-glycero-3-phosphocholine (DMPC) membrane (~50 kJ/mol)^106^. For normal membrane with 0% cholesterol it is much higher (~62 kJ/mol) and increases even more with the increase of CHL content (up to 70 kJ/mol). The difference in the barrier height between the systems with 0% and 15% cholesterol is minimal, but its width is much larger for the 15% system. It is remarkable that the width of the barrier increases mostly in the outer monolayer, which is enriched in cholesterol in the system with 15% CHL content. The PMFs for normal and cancer membranes at 33% CHL content are almost identical.

Figure 6B shows diffusion coefficient of cisplatin in different systems. Again, pure DOPC membrane shows the highest values of *D*. There are no significant differences in *D* between normal membranes with 0%, 15% and 33% of CHL content, which suggests that cholesterol does not influence transversal diffusion of cisplatin. In contrast, there is a clear difference between normal and cancer membranes. Diffusion of cisplatin in the cancer membrane is surprisingly slower especially in the center of the bilayer and in the regions of head groups.

Figure 6C shows the resistance *R* of different membranes to the permeation of cisplatin. The curves follow the shape of the PMFs but their heights are modulated by the difference in diffusion coefficients. The integration of these curves gives the permeabilities presented in Table 3.

**Table 3:** Values of the permeation resistances and permeabilities of cisplatin for different model membranes together with their respective errors.

System	Cholesterol content	Resistance, *R*, *s*⋅*m*^−1^	Permeability, *P*, *m*⋅*s*^−1^
DOPC	0%	4.5 ± 0.1·10^6^	2.2 ± 0.05·10^−7^
Normal	0%	1.15 ± 0.04·10^11^	8.7 ± 0.3·10^−12^
Normal	15%	2.08 ± 0.04·10^11^	4.8 ± 0.1·10^−12^
Normal	33%	5.75 ± 0.08·10^11^	1.74 ± 0.03·10^−12^
Cancer	33%	6.3 ± 0.2·10^12^	1.59 ± 0.06·10^−13^

It is clearly seen that the permeability is the largest for pure DOPC membrane and drops by 4–5 orders of magnitude in the model for plasma membrane with 0% cholesterol. The increase in cholesterol concentration from 0% to 33% decreases the permeability further by another order of magnitude. Finally, symmetrization of the lipid content of the monolayer in the cancer membrane leads to an even larger decrease of permeability. The cancer membrane appears ~11 times less permeable than the normal one.

## Discussion

### Composition of the membranes

The distribution of different lipid species in the membranes of cancer cells is usually discussed in terms of PS exposure, while the distribution of other lipids is rarely considered. This limits available experimental data concerning lipid content of the cancer cell membranes. Taking into account the lack of reliable data we decided to consider the simplest model of the cancer cell membrane where distribution of all lipid species is the same in both monolayers. We are aware that this model is likely to be oversimplified and in reality the membrane is likely to remain somewhat asymmetric. However, a fully symmetric model is the ideal reference system, which could be used to compare the membranes of different cancer cells if their lipid composition and distribution become available.

### The effect of cholesterol

The effect of cholesterol in our simulations is consistent with previous studies which suggest significant ordering and stiffening of the membrane upon an increase in cholesterol concentration. Such stiffening and decrease of the flexibility of the lipid tails decreases the permeability of the membrane to cisplatin by approximately one order of magnitude in comparison with cholesterol-free membrane.

However, there is one puzzling observation in our results: a pronounced asymmetry of cholesterol density distribution in the system with 15% of cholesterol. The question of cholesterol distribution in large heterogeneous and asymmetric systems has only rarely been addressed in MD simulations, but uneven distributions of cholesterol between the leaflets of two model membranes has been reported recently by Ingólfsson *et al*.^4,5^. Close inspection of our system shows that the asymmetry comes from only three cholesterol molecules, which go from the inner to outer monolayer in the course of equilibration. Thus, statistical significance of observed asymmetry is questionable since it could be easily caused by the random motion of few cholesterol molecules rather than systematic reasons. At the same time there is a well-established strong affinity of cholesterol to sphingomyelin which may drive cholesterol molecules to SM-reach outer monolayer of the normal membrane. It is possible to speculate that cholesterol tends to populate the outer leaflet up to a saturation concentration first and then redistributes to the inner leaflet. 15% cholesterol content is likely to be insufficient to saturate the outer leaflet completely which may cause the observed asymmetry. In contrast, 33% cholesterol content is enough for saturation which leads to an equal distribution between the monolayers. One should consider this speculation with care because lateral diffusion and the flip-flop processes should be sampled much better to draw reliable conclusions. It is rather straightforward to test this hypothesis in a series of simulations with different cholesterol distributions but this is beyond the scope of the current work.

### Permeability in comparison to experimental results

It is very important to compare our results with the permeabilities of the membranes of model vesicles and living cells to cisplatin available in the literature. Such comparison could only be done on the semi-quantitative level due to two factors: (1) as it was mentioned in introduction the calculations of permeability using the ISD methodology are known to suffer from inherent limitations (the ISD methodology is likely to provide correct trends when applied to the same ligand in different membranes but it is unlikely to produce values of permeabilities which are quantitatively accurate); (2) the experimental results rarely report permeabilities which could be compared directly to MD data. The majority of the experimental work reports the kinetics of cisplatin permeation through the lipid membranes of cells and artificial liposomes as a single-step first order process^107^. Thus, an effective kinetic constant of permeation, *k* is usually measured in such studies. A relation between permeability and effective kinetic constant is non-trivial in general case and depends on the membrane area, volume of the cell or liposome and membrane heterogeneity^108–110^. Unfortunately there is no experimental research where the area to volume ratio of the cells is determined at the same time as the kinetic constant of cisplatin permeation. That is why in order to estimate the permeability from the available kinetic data we have to use an oversimplified model of spherical cell with radius *r*, which was initially proposed for the liposomes^108–110^:

$$P=\frac{kr}{3}.$$

We considered three experimental data, which allowed the determination of the permeabilities of cisplatin in either cells or artificial membranes (Table 4).

**Table 4:** Values of permeabilities for cisplatin estimated from the literature.

Author	System	Permeability
		P, *m*⋅*s*^−1^
Nierwzicki *et al*.^106^	DMPC bilayer (MD simulations)	6.6710^−7^
Eljack *et al*.^69^	DOPC vesicles ([*Cl*^−^] = 150*mM*	1.0610^−8^
	DOPC vesicles ([*Cl*^−^] = 10*mM*	8.9310^−10^
El-Kareh *et al*.^111^	A2780/CP3 human ovarian resistant cancer	1.8710^−9^
	COLO 205 colon carcinoma	1.2410^−9^
	A2780 human ovarian wild type cells	1.2110^−9^
	CAL 27 head and neck cancer	1.1910^−10^
	MKN45 gastric cancer	1.0110^−10^
	MKN74 gastric cancer	4.9010^−11^
	C32 human melanoma	4.7910^−11^
	G361 human melanoma	2.2110^−11^
Ghezzi *et al*.^72^	MCF-7 breast cancer	6.5710^−11^

In the work of Eljack *et al*.^69^ the permeation of cisplatin in pure DOPC vesicles was studied depending on the concentration of chloride ions in solution (the values for 10 mM and 150 mM external Cl^−^ concentrations are shown in Table 3). Our results for pure DOPC membrane are one order of magnitude higher (~10^−7^ m/s in simulations versus ~10^−8^ m/s in experiment). It is necessary to note that only the counterions necessary to balance the system charge are present in our simulations. We also did not consider the complex interplay of reactions between different cisplatin derivatives which are present in salt solutions and may influence an effective concentration of pristine cisplatin in the experiment. Furthermore, the membranes of our systems are flat while the membranes of real cells and liposomes are significantly curved. We plan to investigate the influence of the curvature on the permeation of the drugs in our future studies but it is clear from multiple indirect evidences that the curvature should affect the permeability. That is why our setup is not directly comparable to experimental conditions which may partially explain observed discrepancies.

The work of Ghezzi *et al*.^72^ reports a time-dependent uptake of cisplatin in breast cancer MCF-7 cell line. Considering the uptake of cisplatin as a first order kinetic process, we fitted the evolution of the intracellular concentration by an exponential function to obtain the kinetic constant (see Supplementary Information and Fig. S6 for the details). The average volume of the cells is estimated at 2 pL in this work. We assumed that the cells are spherical and computed the permeability. The obtained value of ~10^−11^ m/s is 1–2 orders of magnitude higher than our simulation results.

Finally, the work of El-Kareh *et al*.^111^ reports the kinetic constant of cisplatin in different cell lines, which were computed in order to build a pharmacokinetic model for several platinum(II) species. Since the volumes and the area to volume ratios of the studied cells are not reported in this paper we assumed that the cells were spherical and that their volume was the same (2 pL) as in the work of Ghezzi *et al*.^72^ mentioned above. This allowed us to obtain a rough estimate of permeabilities. However, the obtained values are likely to be underestimated since the cells are not spherical and their real area to volume ratio will tend to be higher since real system exhibits complex shape with high membrane curvature.

The only computational work which is directly comparable with the present study is the paper by Nierwzicki *et al*.^106^ where the permeability of cisplatin in a pure DMPC bilayer was reported as 6.6710^−7^ *m*⋅*s*^−1^. This value is of the same order of magnitude as our result for pure DOPC bilayer.

It is possible to conclude that direct quantitative comparison between MD simulations of permeability and the data obtained experimentally on real cell lines remains challenging. The largest uncertainty comes from the fact that the area to volume ratio of the studied cells is not estimated in the experimental studies. Without these data one is restricted to the primitive and inaccurate model of the cell as spherical homogeneous vesicle, which can easily lead to severe underestimation of the permeability by several orders of magnitude. Taking this into account our results look reasonable and consistent.

Although our simulation setup is not directly comparable to experimental conditions and is subject to inherent limitations of the ISD methodology, it is much closer to real membranes than the vast majority of membrane models routinely used in MD studies today. We demonstrated that taking into account realistic lipid composition and cholesterol content brings estimated permeabilities much closer to the experimental values in comparison to simplified membrane models. We expect that accounting for membrane curvature in future works will reproduce experimental results even better. It is possible to speculate that MD simulations of realistic curved and asymmetric membranes with variable cholesterol content allow for reliable semi-quantitative estimation of permeabilities for cisplatin and other platinum drugs.

## Conclusions

In this work we studied the influence of lipid composition and cholesterol content on the permeation of cisplatin through the model membranes of normal and cancer cells.

It is shown that the loss of lipid asymmetry in the cancer membranes leads to decrease their permeability to cisplatin by one order of magnitude in comparison to the asymmetric membranes of normal cells. This effect is caused by slower diffusion of cisplatin in the cancer membrane while the energy barrier of permeation remains the same. It is possible to speculate that this effect may contribute to cisplatin resistance in the cancer cells, which exhibit pronounced loss of lipid asymmetry.

The change of cholesterol molar ratio from 0% to 33% also decreases the permeability of the membrane by approximately one order of magnitude. Thus, changes of cholesterol content in cancer cell membranes may significantly influence their permeability to cisplatin.

It is also shown that the single-component DOPC membrane is a very poor model for cisplatin permeation in real cells since its permeability is 5–6 orders of magnitude higher than one of the membranes with a more realistic lipid composition.

We also conclude that direct quantitative comparison between MD simulations of permeability and the data obtained experimentally on real cell lines remains challenging due to the lack of models, which take into account complex shape of the cells, and uncertainty related to chemical modifications of cisplatin in solution.

## Supplementary information

Supporting informations
