# Supplementary material for: The asymmetry of plasma membranes and their cholesterol content influence the uptake of cisplatin
Source: Sci Rep. 2019 Apr 4;9:5627. doi: 10.1038/s41598-019-41903-w (PMC6449338; doi:10.1038/s41598-019-41903-w)
Supplement: Supplementary file 1 — Supporting informations [file 41598_2019_41903_MOESM1_ESM.docx]

**The asymmetry of plasma membranes and their cholesterol content influence the uptake of cisplatin**

T. Rivel, C. Ramseyer, S. O. Yesylevskyy

**Supplementary information**

*Overexposure of phosphatidylserine (PS) in tumorigenic cells– a brief review of the literature*

**Table S1.** Overview of the relevant publications referring to a quantitative increase in PS exposure in the extracellular leaflet of the plasma membrane of tumorigenic cells. A group of publications addressing specially the overexpression of PS (and possibly the phosphatidylethanolamine) in the tumoral vasculature cells has been omitted. If this was possible, the relative increase in proportion of PS in the extracellular leaflet in a tumoral cell versus a reference one has been indicated. The cited papers are the work of Connor *et al.*[^1^](#_ENREF_1), Utsugi *et al.*[^2^](#_ENREF_2), Rao *et al.*^[3](#_ENREF_3)^, Sugimura *et al.*^[4](#_ENREF_4)^, Cichorek *et al.*^[5](#_ENREF_5)^, Schröder-Borm *et al.*^[6](#_ENREF_6)^, Kirszberg *et al.*^[7](#_ENREF_7)^ and Riedl *et al.*^[8](#_ENREF_8)^.

*
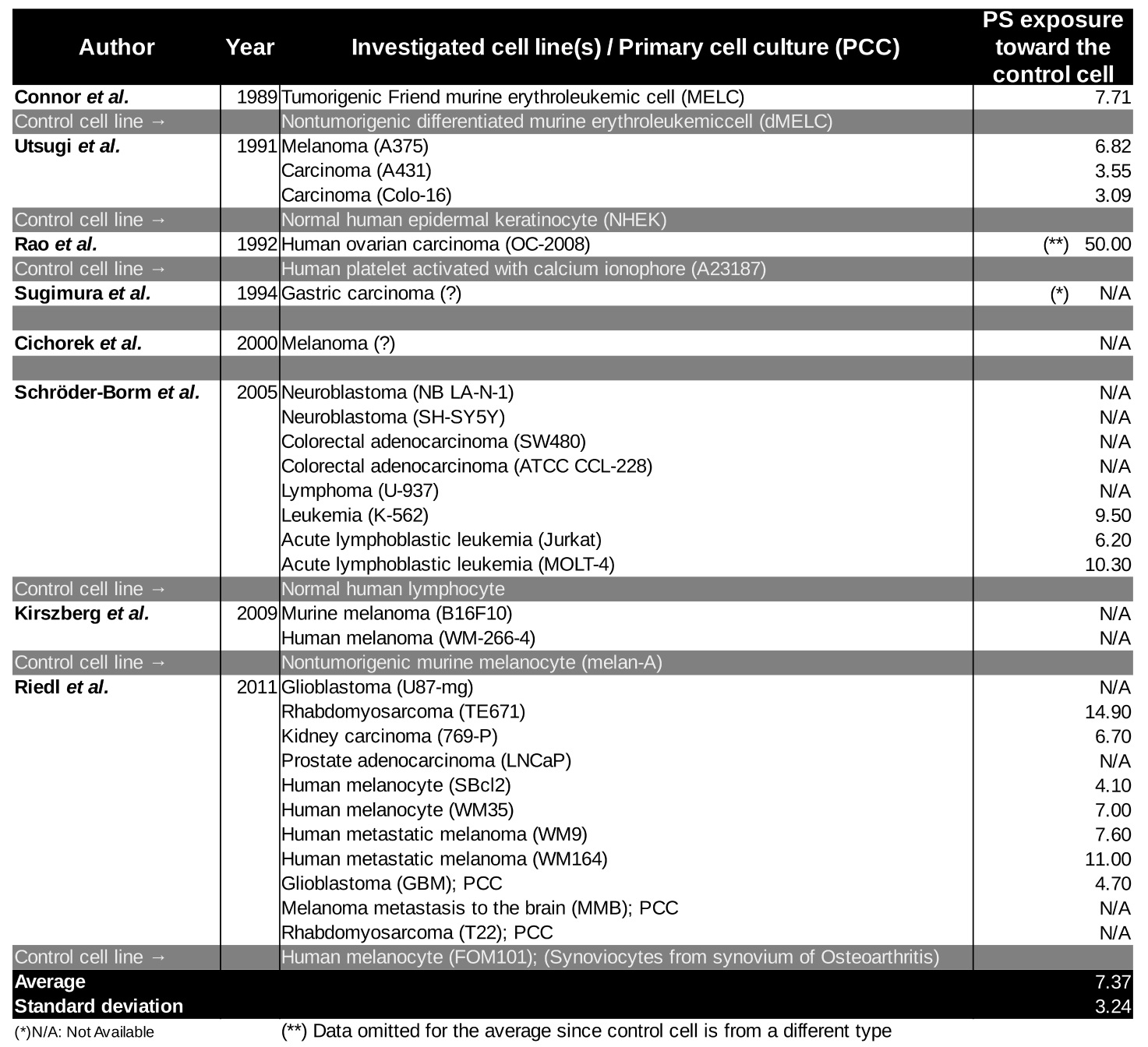
*

*Membrane construction setup*

In this work we used the method for keeping global membrane shape by means of artificial dummy particles, which was used in our previous works[^9^](#_ENREF_9)^,^[^10^](#_ENREF_10). The idea is to put the membrane between two repulsive surfaces of artificial particles - the walls - which restrict the global shape of membrane but which do not affect lateral dynamics of individual membrane lipids (Fig S1).

The walls are composed of independent non-interacting beads which consist of an anchor and shell particles. The anchor is a dummy particle which is fixed in absolute coordinates and which does not interact with any other particles in the system. The shell particle is connected to an anchor by a harmonic bond of zero length with the force constant of 10 kJ·mol^-1^·nm^-2^. The shell particles selectively interact with the carbon atoms of the lipid acyl tails by Van der Waals interactions only. Parameters of this Lennard-Jones interaction are *σ*=0.85 nm and *ε*=1·10^-5^ J/mol which means that the potential is purely repulsive within the short-range cut-off *r_c_*=0.8 nm widely adopted in Amber force field[^11^](#_ENREF_11). Such a setup eliminates unwanted direct interactions of the lipid atoms with fixed anchor particles and allows the walls to be positioned precisely at the same time by moving anchors to the desired position.

The walls are initially at the level of lipid head groups of each monolayer of the flat bilayer at a distance of 5 nm from each other. The dummy beads are arranged in a rectangular grid in the plane of the wall with a spacing of ~0.51 nm.

The shell particles of the walls are impermeable for the hydrophobic lipid tails but completely transparent for all other atoms. Thus, they restrict the general shape of the membrane hydrophobic core effectively without influencing the lateral diffusion of the lipids or the dynamics of lipid head groups, cholesterol, ions and water molecules. The distance between the walls and the parameters of repulsive Van der Waals interactions are adjusted empirically in order to minimize the influence of the walls on the membrane structure and properties. The density profiles of various chemical groups of the lipids were compared for planar bilayers with and without the walls. The results obtained show that the changes in the structure of the bilayer introduced by the walls are barely visible and can be safely neglected[^9^](#_ENREF_9)^,^[^10^](#_ENREF_10).


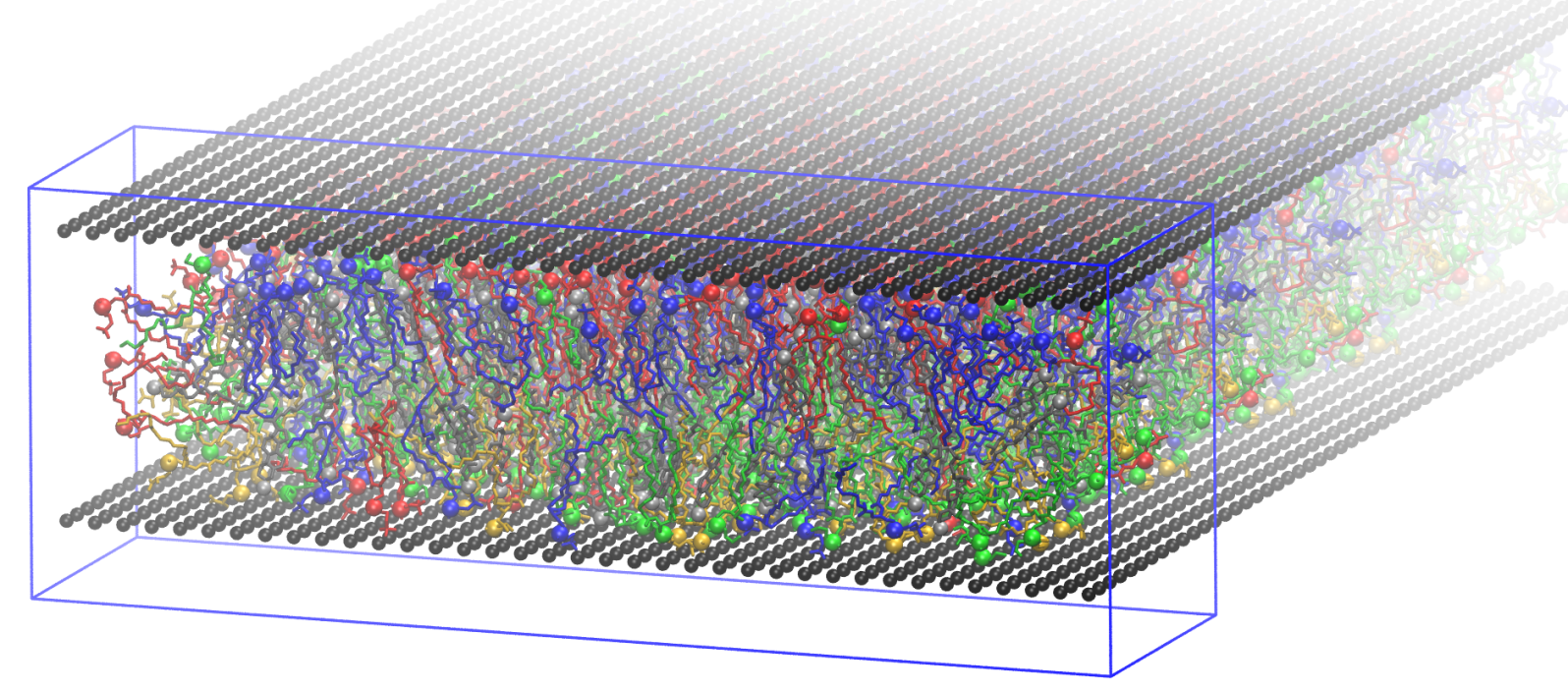


**X**

**Z**

**Y**

**Figure S1.** Perspective view of the simulated “normal membrane” system. The wall particles are shown as black spheres. PC lipids are blue, SM are red, PE are green and PS are yellow. Cholesterol molecules are gray. Head groups of the lipids the cholesterol are shown in as spheres. Four periodic images of the system are shown in the Y direction. The blue frame shows the simulation box.

*Treatment of the bicelle caps*


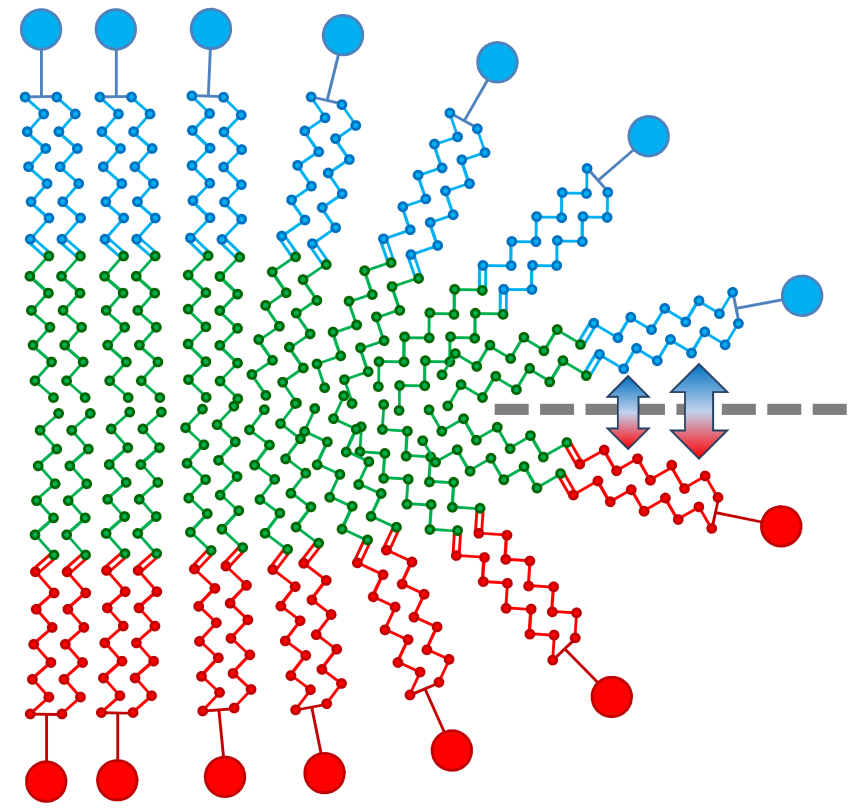


Figure S2. Scheme of the bicelle cap which prevents mixing of the lipids from different monolayers. Distal parts of the tails (below the double bond), which interact normally, are shown in green. Proximal parts of the tails and head groups of lipids from different monolayers are shown in blue and red respectively. There is an additional artificial Van der Waals repulsion between blue and red atoms (shown by the arrows) which prevents mixing of the lipids from different monolayers. Approximate boundary between the monolayers is shown by gray dashed line.

*Sampling lateral heterogeneity of the membrane*

In order to check if the lateral heterogeneity of the membrane is sampled sufficiently we analyzed the local lipid environment of each cisplatin molecule present in the system during the simulations. The lipid molecule was considered to be in the local vicinity of the ligand if any of its atoms was closer than 0.8 nm to any atom of the ligand. The data were averaged over the last 10 ns of all umbrella sampling windows used for the computation of the PMFs of cisplatin.


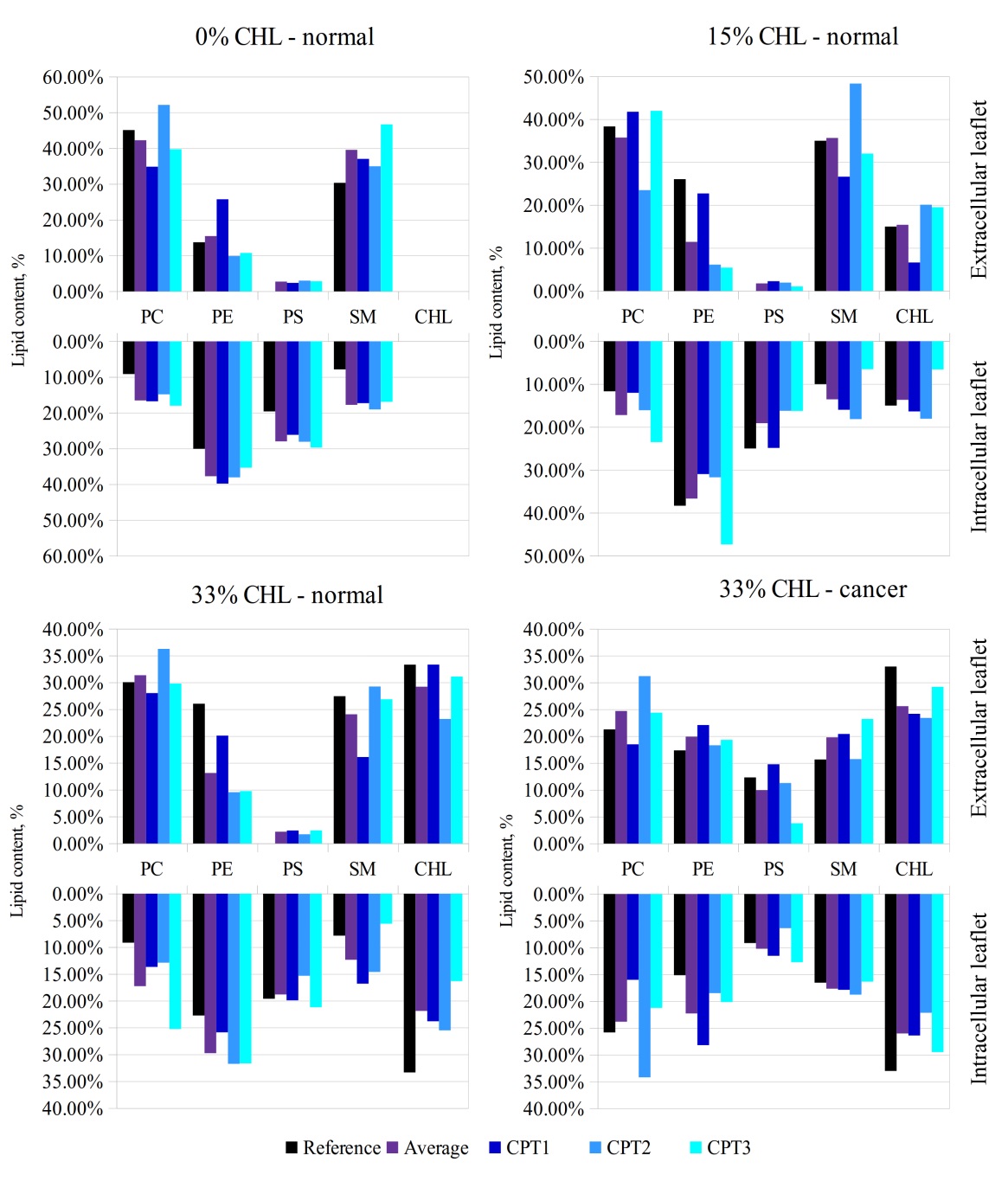


**Figure S3.** Local membrane composition in the vicinity of individual ligands and their average in comparison to overall composition of the corresponding membrane leaflets (reference).

The local membrane composition felt by each individual CPT molecule follows the overall membrane composition. However, some differences exist in between different CPT molecules due to the lateral inhomogeneity of our model membranes.

*Convergence of the PMF computations*

The PMF computations where achieved by means of umbrella sampling. The umbrella sampling runs where carried out up to the moment a visual convergence was reached. The figure S1 shows the convergence graph in the case of the cisplatin through the cancer membrane. In this figure four PMF are plotted which are taking the last 5 ns of runs of different lengths.


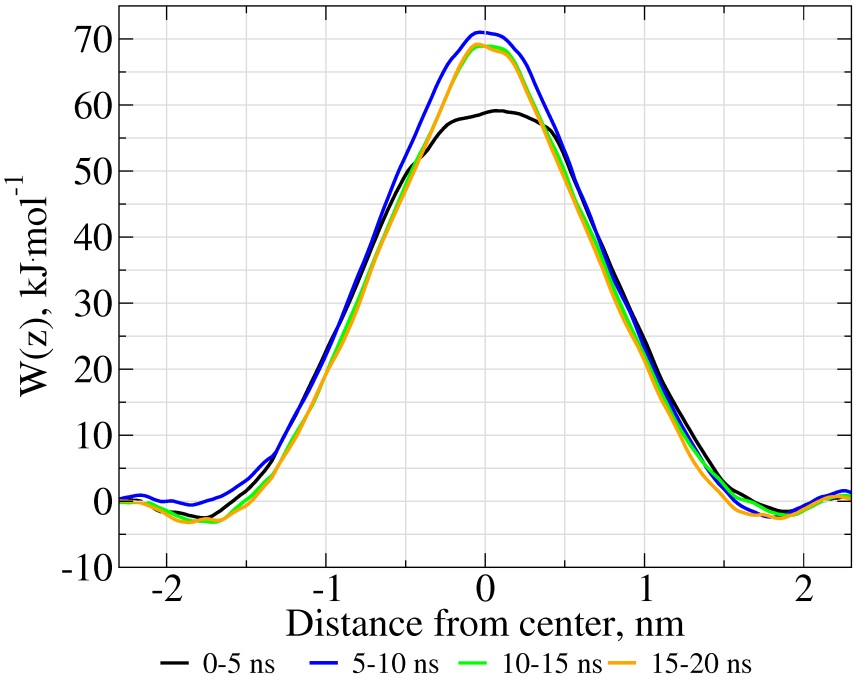


**Figure S4.** PMFs convergence in the case of the cisplatin through the cancer model membrane.

*Remark on the simulation time step*

It is necessary to note that a time step of 1fs without converting the bonds to rigid constraints is usually considered to be too large – due to the too high frequencies of vibration of certain bonds – for insuring the energy conservation. However, our systems were never used without thermostat (like in the NVE ensemble), thus, this issue does not affect the stability of the simulations. In our systems, the NPT ensemble is always used, which eliminates any systematic energy drifts due to the fact that the energy conservation is not ideal during the integration of certain bonds.

*Standard deviations of diffusion coefficients*


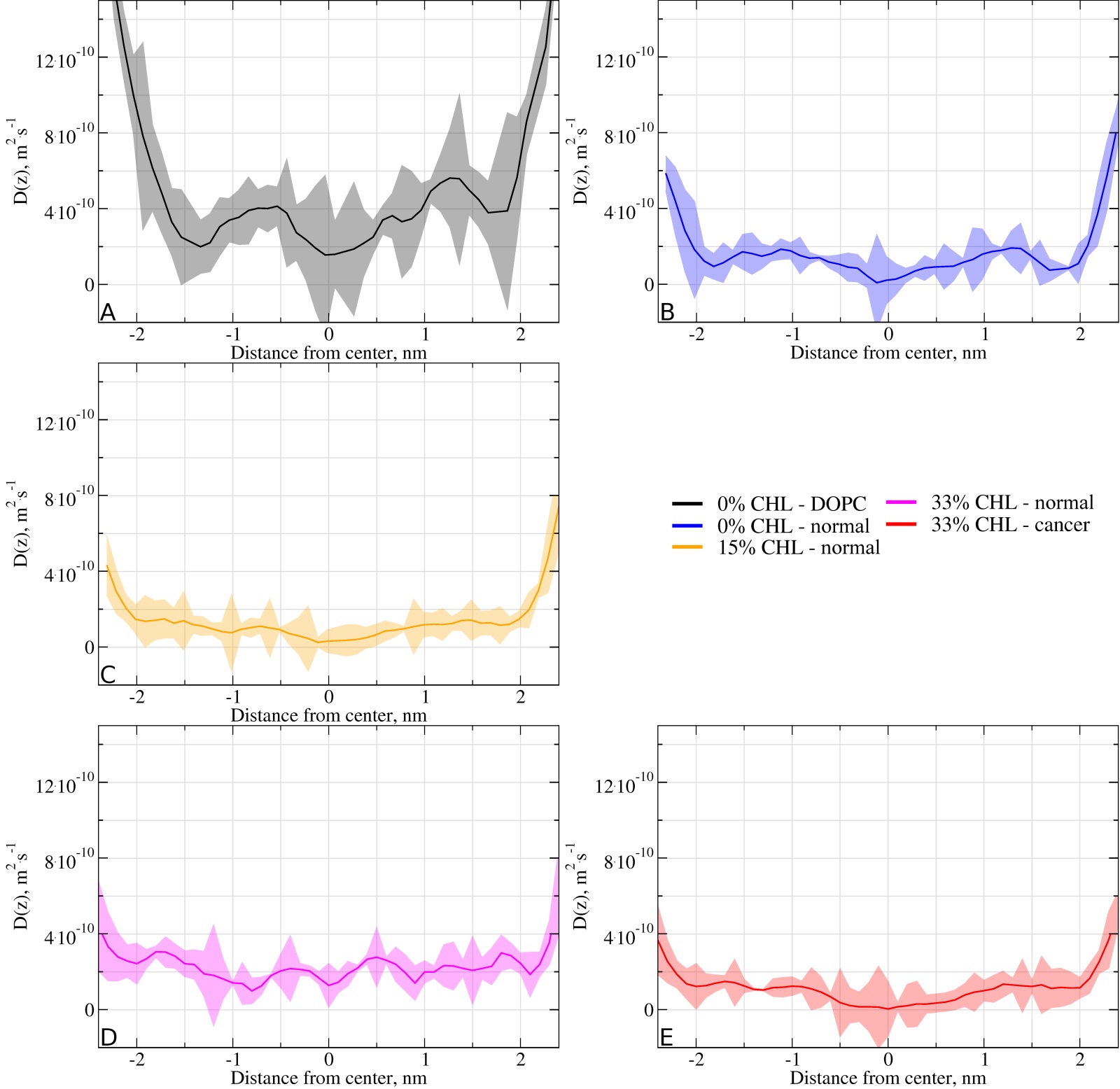


**Figure S5.** Diffusion coefficients of cisplatin and their standard deviations.

*Computing the errors of membrane resistance and permeability*

The errors of resistances and permeabilities were estimated using the error propagation formalism based on the standard deviations of *W*(*z*) and of *D*(*z*). For the resistance *R* at particular point *z* we get:

$\sigma\left( R\left( z \right) \right)=R(z)\sqrt{\left( \frac{\sigma\left( W\left( z \right) \right)}{k_{B}T} \right)^{2}+\left( \frac{\sigma\left( D\left( z \right) \right)}{D\left( z \right)} \right)^{2}}$,

where $\sigma$ is the standard deviation of *R*. In order to get global resistance one has to integrate *R*(*z*) over *z.* Since the integral is approximated by a discrete sum the following expression for the standard deviation of *R* could be derived:

$\sigma\left( R \right)=\sqrt{\sum_{i} \left( R_{i}{dz}_{i} \right)^{2}}$,

where *i* denotes one discrete value and *dz_i_* is the size of *i*-th integration step. Finally, the error of *P* is computed as:

$\sigma\left( P \right)=P\frac{\sigma(R)}{R}$.

*Obtaining kinetic constant from the time-dependent platinum uptake analysis*

In order to obtain the kinetic constant from the work from Ghezzi et al., we considered that the uptake is a first-order reaction:

${Pt}_{extra}\rightleftharpoons{Pt}_{intra}$,

where ${Pt}_{extra}$ and ${Pt}_{intra}$ are the platinum species concentrations outside and inside the cell respectively. One can write the following kinetic equation:

$-\frac{d{Pt}_{extra}}{dt}=k{Pt}_{extra}$.

The straightforward solution of such first order differential equation is:

${Pt}_{extra}\left( t \right)={Pt}_{extra}^{0} e^{-k t}$.

where ${Pt}_{extra}^{0}$ is initial extracellular concentration at time $t=0$.

The intracellular concentration is:

${Pt}_{intra}\left( t \right)={Pt}_{extra}^{0} \left( 1-e^{-k t} \right)$.

This equation was used to fit experimental data of Ghezzi et al. (Fig. S5). A value for k of 0.091 h^-1^ was found to provide the best fit (fitting R^2^ value of 0.97).


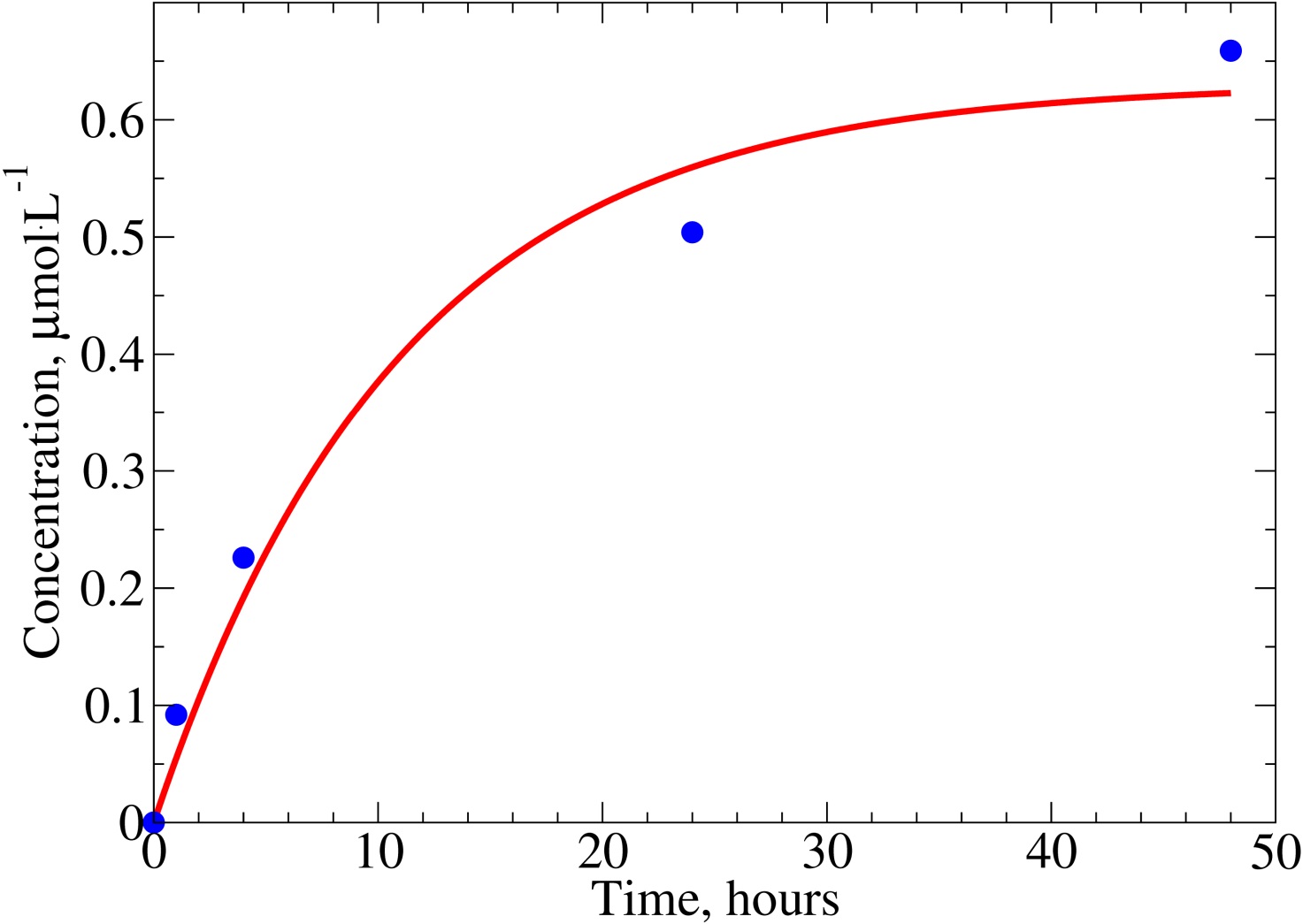


**Figure S6.** Fitting of the experimental values obtained by Ghezzi *et al.*[^12^](#_ENREF_12) by analytical formula assuming first-order cisplatin uptake reaction.

**References**

1 Connor, J., Bucana, C., Fidler, I. J. & Schroit, A. J. Differentiation-dependent expression of phosphatidylserine in mammalian plasma membranes: quantitative assessment of outer-leaflet lipid by prothrombinase complex formation. *PNAS* **86**, 3184-3188, doi:10.1073/pnas.86.9.3184 (1989).

2 Utsugi, T., Schroit, A. J., Connor, J., Bucana, C. D. & Fidler, I. J. Elevated Expression of Phosphatidylserine in the Outer Membrane Leaflet of Human Tumor Cells and Recognition by Activated Human Blood Monocytes. *Cancer Res* **51**, 3062-3066 (1991).

3 Rao, L. V. M., Tait, J. F. & Hoang, A. D. Binding of annexin V to a human ovarian carcinoma cell line (OC-2008). Contrasting effects on cell surface factor VIIa/tissue factor activity and prothrombinase activity. *Thrombosis Research* **67**, 517-531, doi:10.1016/0049-3848(92)90013-z (1992).

4 Sugimura, M., Donato, R., Kakkar, V. V. & Scully, M. F. Annexin V as a probe of the contribution of anionic phospholipids to the procoagulant activity of tumour cell surfaces. *Blood Coagul. Fibrinolysis* **5**, 365-373 (1994).

5 Cichorek, M., Kozłowska, K., Witkowski, J. M. & Zarzeczna, M. Flow cytometric estimation of the plasma membrane diversity of transplantable melanomas, using annexin V. *Folia Histochem. Cytobiol.* **38**, 41-43 (2000).

6 Schröder-Borm, H., Bakalova, R. & Andrä, J. The NK-lysin derived peptide NK-2 preferentially kills cancer cells with increased surface levels of negatively charged phosphatidylserine. *FEBS Letters* **579**, 6128-6134, doi:10.1016/j.febslet.2005.09.084 (2005).

7 Kirszberg, C. *et al.* Simultaneous tissue factor expression and phosphatidylserine exposure account for the highly procoagulant pattern of melanoma cell lines. *Melanoma Research* **19**, 301-308, doi:10.1097/CMR.0b013e32832e40fe (2009).

8 Riedl, S. *et al.* In search of a novel target — Phosphatidylserine exposed by non-apoptotic tumor cells and metastases of malignancies with poor treatment efficacy. *Biochimica et Biophysica Acta (BBA) - Biomembranes* **1808**, 2638-2645, doi:10.1016/j.bbamem.2011.07.026 (2011).

9 Cherniavskyi, Y. K., Ramseyer, C. & Yesylevskyy, S. O. Interaction of C60 fullerenes with asymmetric and curved lipid membranes: a molecular dynamics study. *Physical Chemistry Chemical Physics* **18**, 278-284, doi:10.1039/c5cp05838d (2016).

10 Yesylevskyy, S. O., Rivel, T. & Ramseyer, C. The influence of curvature on the properties of the plasma membrane. Insights from atomistic molecular dynamics simulations. *Scientific Reports* **7**, 16078, doi:10.1038/s41598-017-16450-x (2017).

11 Hornak, V. *et al.* Comparison of multiple Amber force fields and development of improved protein backbone parameters. *Proteins: Structure, Function, and Bioinformatics* **65**, 712-725, doi:10.1002/prot.21123 (2006).

12 Ghezzi, A., Aceto, M., Cassino, C., Gabano, E. & Osella, D. Uptake of antitumor platinum(II)-complexes by cancer cells, assayed by inductively coupled plasma mass spectrometry (ICP-MS). *Journal of Inorganic Biochemistry* **98**, 73-78, doi:10.1016/j.jinorgbio.2003.08.014 (2004).
